# Supplementary material for: The interactome of intact mitochondria by cross-linking mass spectrometry provides evidence for coexisting respiratory supercomplexes
Source: Mol Cell Proteomics. 2017 Dec 8;17(2):216–32. doi: 10.1074/mcp.RA117.000470 (PMC5795388; doi:10.1074/mcp.RA117.000470)
Supplement: Supplemental Data [file supp_RA117.000470_133922_0_supp_23978_fzffwf.zip › spectra_annotation/mito_spectra_annotation/121-1-1-1-3-1.pdf]

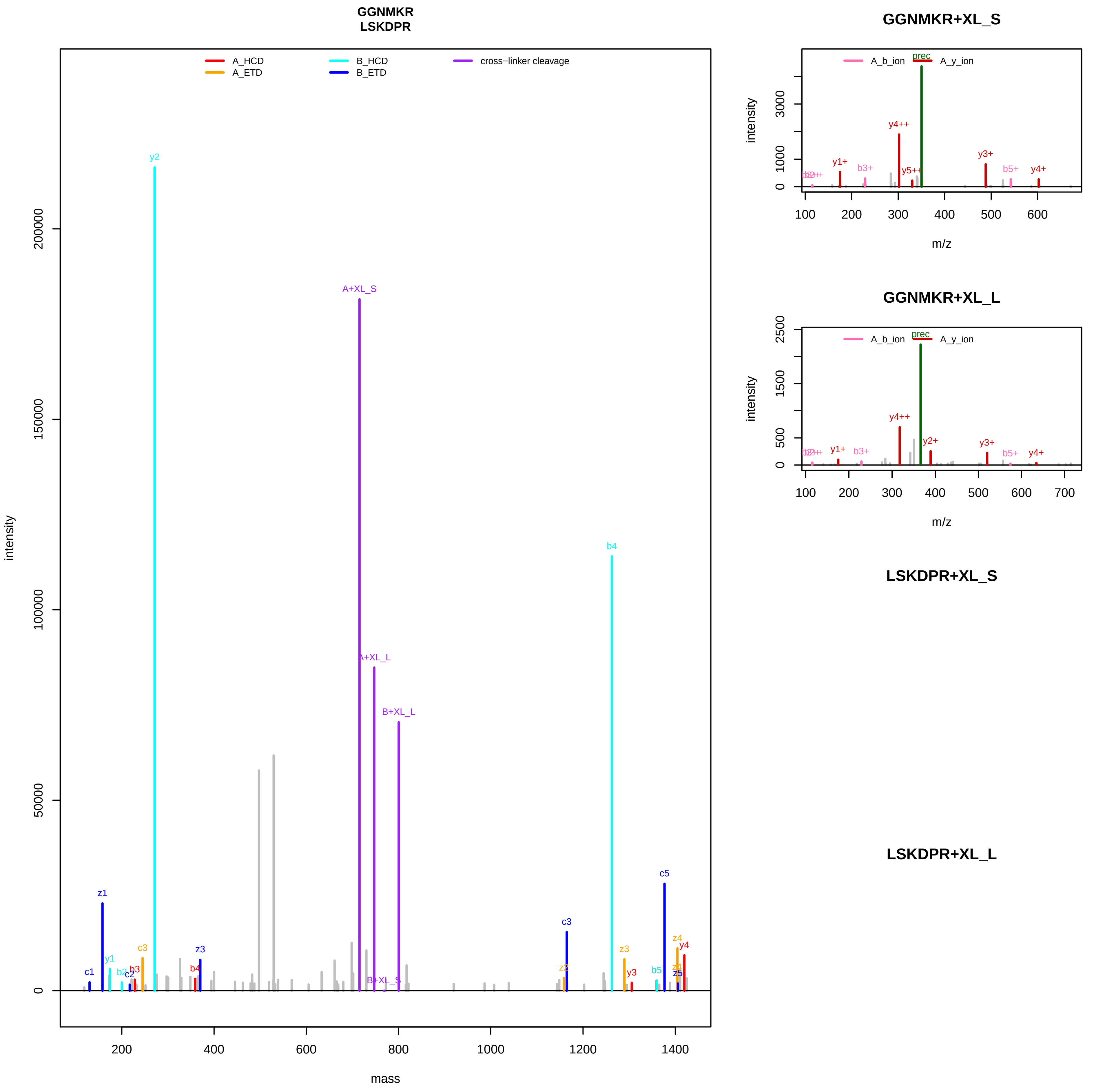
GGNMKR
LSKDPR
A_HCD
A_ETD
B_HCD
B_ETD
cross−linker cleavage
intensity
mass
y2
A+XL_S
A+XL_L
B+XL_L
B+XL_S
b4
c5
c3
z1
c1
y1
b2
c2
b3
c3
z3
b4
z2
z3
y3
b5
z4
y4
z5
GGNMKR+XL_S
A_b_ion
A_y_ion
prec
intensity
m/z
y4++
y1+
b3+
y5++
y3+
b5+
y4+
GGNMKR+XL_L
y2+
LSKDPR+XL_S
LSKDPR+XL_L
